# Pacemaker Implantation in Patient With Persistent Left Superior Vena Cava and Absent Right Superior Vena Cava

**DOI:** 10.7759/cureus.7980

**Published:** 2020-05-05

**Authors:** Syed Rafay Ali Sabzwari, James Kimber, Sara A Godil, Waqas Khan, Jawad Mir

**Affiliations:** 1 Cardiology, Lehigh Valley Health Network, Allentown, USA; 2 Internal Medicine, Lehigh Valley Health Network, Allentown, USA; 3 Internal Medicine, Army Medical College, Rawalpindi, PAK

**Keywords:** pacemaker, persistent left superior vena cava, absent right superior vena cava

## Abstract

Persistent left superior vena cava (LSVC) is an asymptomatic congenital heart disease. It is usually found incidentally on imaging, during central line placements or while undergoing electrophysiological procedures. We present a case of a 91-year-old female who initially presented with seizures and was diagnosed with tachy-brady syndrome. She was planned to undergo dual-chamber permanent pacemaker placement. However, during the procedure, she was incidentally found to have an LSVC without a right superior vena cava. Due to challenging anatomy, her pacemaker was changed to a single-chamber atrial lead pacemaker. This case highlights the clinical implications of this unusual structural anomaly, technical difficulties that arise alongside and solutions on how to overcome these issues in the context of pacemaker implantation.

## Introduction

Persistent left superior vena cava (LSVC) is an abnormality affecting approximately 0.5% of the general population and 4.5%-11% of patients with congenital heart disease [1-4]. In embryogenesis, the vena cava is formed at the sinus venosus, where pairs of cardinal veins merge with the vitelline and umbilical veins. The right cardinal vein becomes the right superior vena cava (SVC), and typically the left cardinal vein involutes and disappears as a result of extrinsic compression (by the left atrium and hilum). When the left cardinal vein persists, it leads to persistence of an LSVC. Most commonly, the left-sided SVC enters the coronary sinus (CS) with subsequent drainage into the right atrium, although approximately 8% of the cases drain into a left atrium, which may be associated with a right to left shunt. In 80% of LSVC cases, a right SVC is present [1]. However, when the right SVC is absent (prevalence of 0.07%-0.13%), the venous return to the superior system is shunted through the CS, which becomes dilated [3]. This condition is tolerated well, often identified incidentally during imaging or procedures. This case highlights the technical challenge complicating device insertion when there is anomalous venous anatomy

## Case presentation

**Figure 1:**
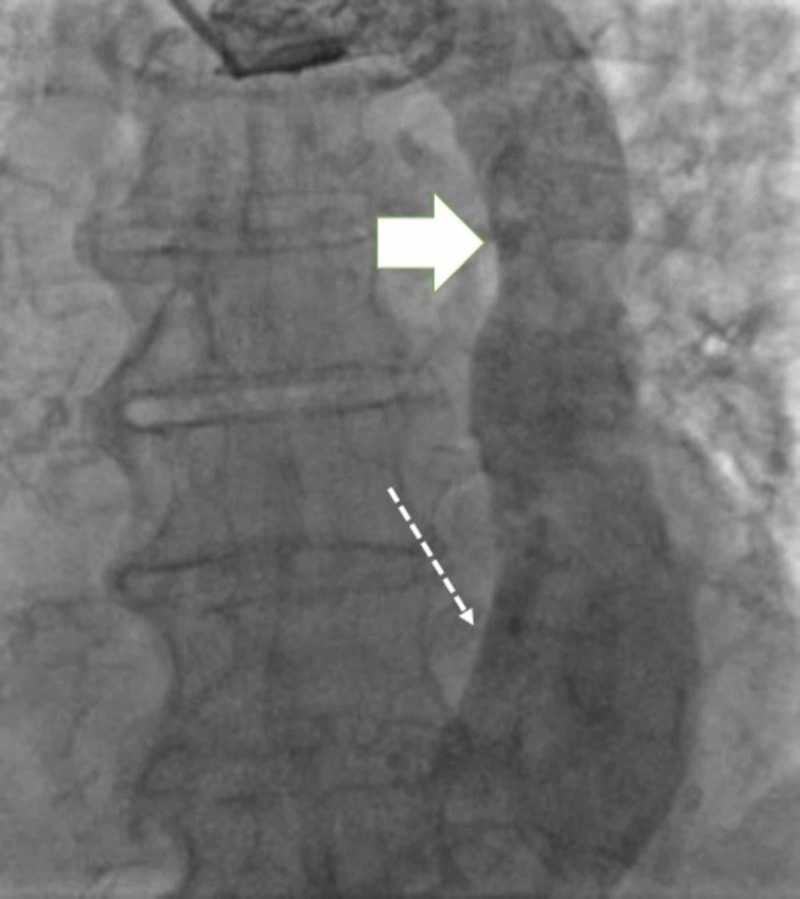
Venography demonstrating LSVC (solid arrow) draining to dilated CS (dotted arrow) CS: coronary sinus, LSVC: left superior vena cava

A 91-year-old female was admitted for tremors/convulsions with focal seizure. While hospitalized, she was found to have tachy-brady syndrome with heart rates exceeding 200 beats per minute, accompanied by symptomatic seven-second pauses. She was referred for dual-chamber pacemaker implantation. During pacemaker insertion, left upper extremity venography was performed, which revealed persistent LSVC with drainage into the CS (Figure 1).

**Figure 2:**
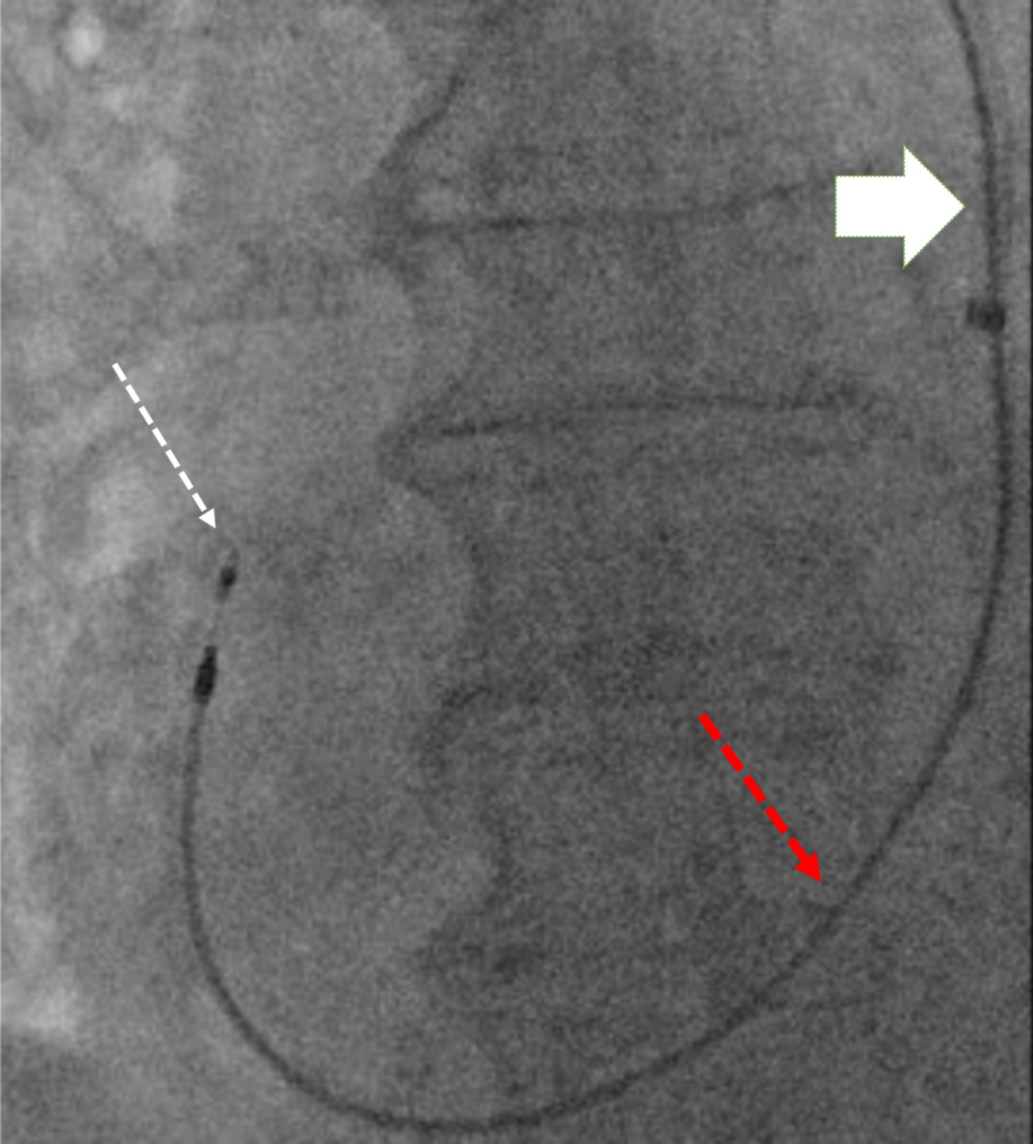
Pacer lead passing from LSVC (white solid arrow) to right atrium (white dotted arrow) through the CS (red dotted arrow) CS: coronary sinus, LSVC: left superior vena cava

**Figure 3:**
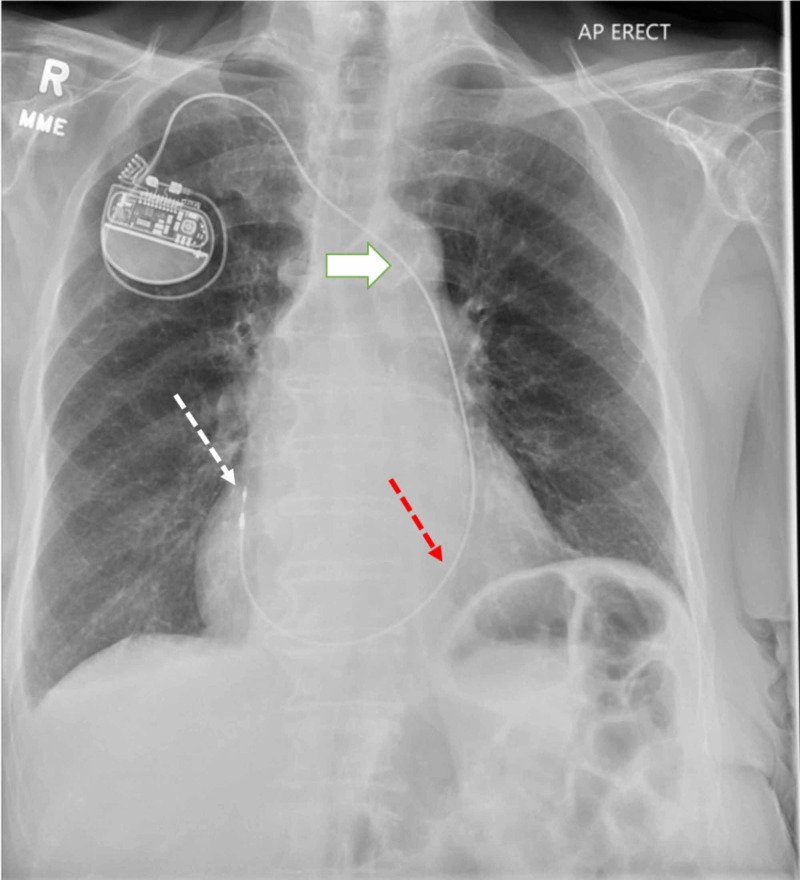
Chest radiograph with a pacer lead coursing through LSVC (white solid arrow), CS (red dotted arrow), and terminating in RA (white dotted arrow) CS: coronary sinus, LSVC: left superior vena cava, RA: right atrium

Subsequently, a right axillary vein was accessed for lead placement, and a right ventricular lead (used as a surrogate instead of right atrial lead due to its longer length) was inserted, passing to the right atrium via the CS, and confirming the absence of a right SVC (Figures 2, 3). 

**Figure 4:**
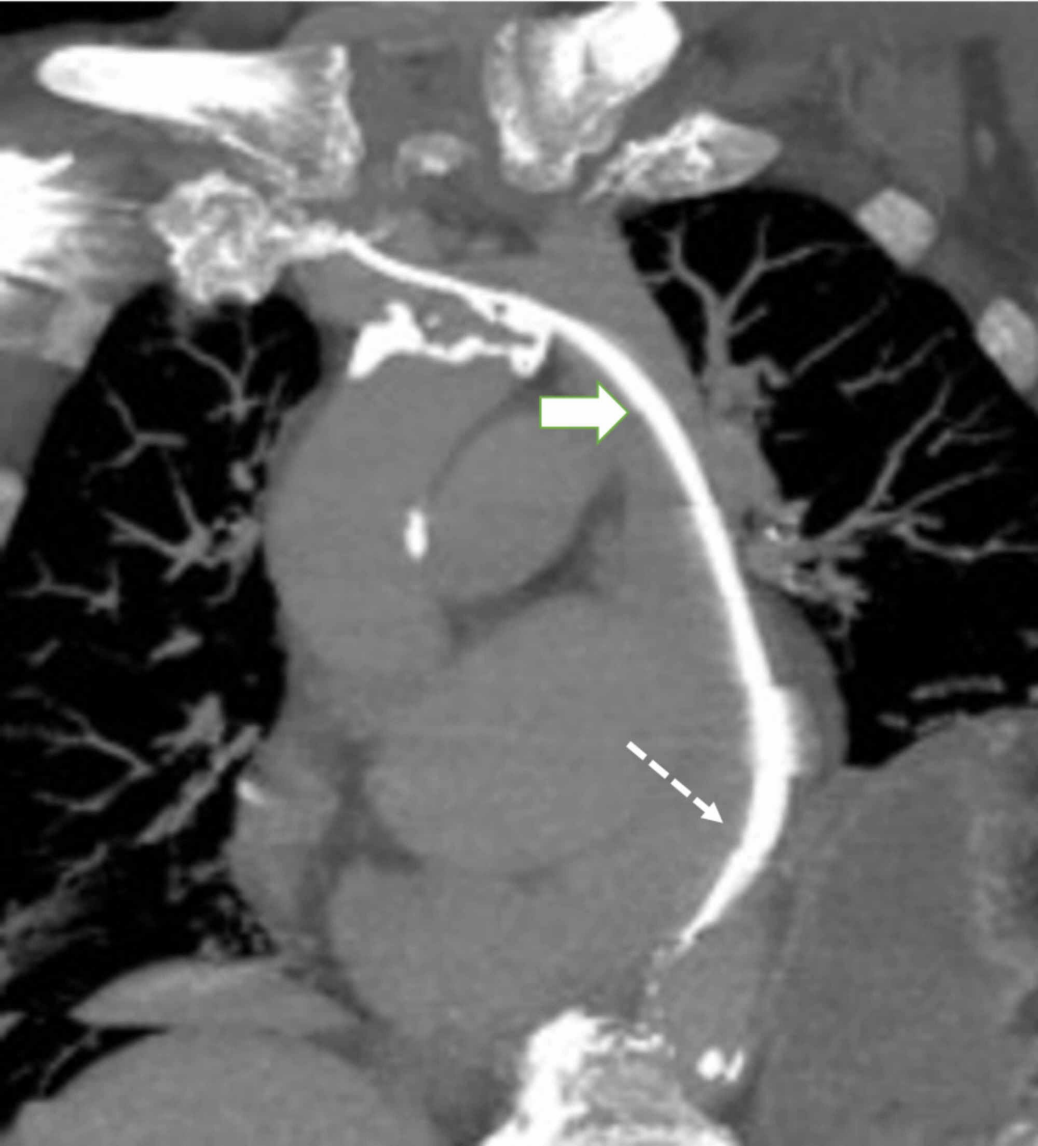
CT of the chest with subtraction imaging demonstrating the course of pacer lead through persistent LSVC (solid arrow) and CS (dotted arrow) CS: coronary sinus, LSVC: left superior vena cava

Attempts to navigate the lead across the tricuspid valve were unsuccessful, and the procedure was modified for single-chamber atrial pacer lead only as the patient had preserved atrioventricular conduction. Following the insertion of a right atrial lead, a CT scan confirmed this anatomic variant, and demonstrated pacemaker lead coursing through persistent left SVC with tip in the right atrial appendage (Figure 4).

## Discussion

Persistent LSVC occurs in 0.5% of the general population and more frequently in patients with congenital heart disease [1-5]. Some congenital abnormalities (atrial septal defect, cor triatriatum and mitral atresia) are associated with persistent LSVC, in part because of decreased compression between the left atrium and hilum in development. When unknown, LSVC may complicate central venous catheter and device placement. Pacer lead insertion may be technically challenging due to misalignment of the CS ostium in relation to the tricuspid valve. Historically, operators have overcome these complications by formation of a pacer wire loop prior within the right atrium, utilizing CS guide catheters for directional support, or by insertion of a pacer lead into the CS branches [6-8].

Although preprocedural 12-lead ECG and chest x-ray may elucidate many congenital abnormalities, persistent LSVC can go unnoticed and may be discovered incidentally during invasive procedures [9]. In the case of pacer insertion, it is common to perform transthoracic echocardiography prior to procedure. When a large CS is identified, agitated saline injected into the left arm visualized traversing through the CS can be used to confirm the diagnosis of LSVC. Alternatively, this anomaly can be identified by CT or MRI [10].

While lead insertion is usually possible, anomalous venous circulation may lead to complex procedure with prolonged radiation exposure. Preprocedural identification may allow operators to plan for alternate strategies of device insertion, e.g. left ventricular lead through CS, epicardial leads, or leadless devices.

## Conclusions

Permanent pacemaker placement may be complicated by anatomic variants involving SVC. Anatomic variation may necessitate the placement of transinferior vena cava or epicardial lead implant if otherwise unsuccessful. Although pacemaker lead insertion via the CS has been associated with complications including thrombosis of CS, this has been successfully done as illustrated in our case. 

## References

[1] Kumar SSNR, Dhulipala S, Subhrahmanyam AH, Pisharath MKS (2005). Absent right superior vena cava. Indian J Thorac Cardiovasc Surg.

[2] Motwani M, Cassidy C, Clarkson P (2010). An alternative technique for implantation of a dual chamber pacemaker via a persistent left superior vena cava using a coronary sinus guiding catheter. J Cardiol Cases.

[3] Petrac D, Radeljic V, Pavlovic N, Manola S, Delic-Brkljacic D (2013). Persistent left superior vena cava in patients undergoing cardiac device implantation: clinical and long-term data. Cardiol Res.

[4] Sonou A, Hounkponou M, Codjo L (2017). Detection of a left superior vena cava during a pacemaker implantation in cotonou. Case Reports Cardiol.

[5] Tak T, Crouch E, Drake GB (2002). Persistent left superior vena cava: incidence, significance and clinical correlates. Int J Cardiol.

[6] Sundhu M, Syed M, Gul S, Saqi B, Mosteller R (2017). Pacemaker placement in persistent left superior vena cava. Cureus.

[7] Girerd N, Gressard A, Berthezene Y, Lantelme P (2009). Persistent left superior vena cava with absent right superior vena cava: a difficult cardiac pacemaker implantation. Int J Cardiol.

[8] Kumar S, Moorthy N, Kapoor A, Sinha N (2012). A challenging dual chamber permanent pacemaker implantation in persistent left superior vena cava with absent right superior vena cava. J Cardiol Cases.

[9] Kimber J, Sabzwari S, Mann K, Ayele H, Bozorgnia B, Dusaj R (2019). Pacemaker implantation in patient with persistent left superior vena cava (SVC) with absent right SVC. J Am Coll Cardiol.

[10] Cha EM, Khoury GH (1972). Persistent left superior vena cava. Radiologic and clinical significance. Radiology.

